# Editorial: The Impact of Online Addiction on General Health, Well-Being and Associated Societal Costs

**DOI:** 10.3389/fpubh.2021.676498

**Published:** 2021-04-01

**Authors:** Georgios D. Floros, Konstantinos Ioannidis

**Affiliations:** ^1^2nd Department of Psychiatry, School of Medicine, Aristotle University of Thessaloniki, Thessaloniki, Greece; ^2^Department of Psychiatry, University of Cambridge, Cambridge, United Kingdom

**Keywords:** online addiction, gaming addiction, problematic internet use, well-being, health impact

Addictive online behaviors have come under increasing research scrutiny in the past two decades, culminating in the recent inclusion of gaming disorder as a distinct entity in the 11th Revision of the International Classification of Diseases (ICD-11) (1). Beyond gaming, a number of distinct problematic behaviors have been linked with poor mental health (2, 3), academic underachievement and vocational struggles (4), and described in distinct geographical areas and across cultural settings (5–7). The widespread adoption of online applications for communication and recreation has caused the wider public health discussion and concern (2). While our over-reliance on technology grows, so does the possibility that exposure to online applications can have pervasive health consequences, particularly in a time when societies have specifically restructured to fight against the COVID-19 pandemic: by embracing the online milieu for work, relationships, and entertainment (8).

Although treatment options are available for some time now (9, 10), what has been less understood has been how online addiction and technology overuse may lead to significant consequences to society, including but not exhaustively, impact on quality of life, productivity and collateral costs to the health system. In this special issue, the wider impact to the general health and well-being of the affected individuals was put under examination with the goal of exploring the effects of online addictive behaviors, including but not limited to, online gaming and social media, to the burden on the individual's general health and well-being. Of special interest was how this burden carries over to the macro level in the workplace, the educational system and also the health system, with general productivity loss, missed opportunities at work and education, and increased use of the health services. We were pleased to receive a number of high-quality studies reporting on this burden and helping the exploration of cost-effectiveness of treatment modalities and highlight the need for investment in health services tailored to population needs.

It is important to note that online addiction is not an artificial construct that originated from psychological research but rather was a concept that sought to include complaints from patients and their families in order to better understand their source and provide help; in this respect a very important contribution in this Research Topic is the one made by Gjoneska et al. who present a conceptual framework and a template for citizen involvement in research on online addiction; the authors highlight four ethical aspects for citizen involvement, driven by community case studies that were conducted in six European countries.

With regards to the impact of online addiction on general health, Mylona et al. examined in a literature review the effects of gaming disorder in eyesight and eye pathology, including eye strain. Prolonged engagement with screens and digital media can have direct strenuous impact on the visual system (11, 12), possibly related to the necessity for prolonged near-term adaptation, but also related to mechanical consequences of prolonged attention to the screen e.g., dry eye syndrome. Mylona et al. further comment on sequelae of poor posture and prolonged physical immobility which are commonly reported.

In an original investigation of problematic usage of the internet, Lam investigated the effects of parental depression on adolescent internet addiction using structural equation modeling. He focused on the by-gender relationships between parental mental health (depression and internet addiction) and demonstrated mediation of adolescent mental stress on the relationship between parental depression and adolescent internet addiction; those mediating effects were stronger in the same gender parent-adolescent dyads.

In another original prospective investigation, Ueno et al. explored the impact on online addiction in a specific work setting, that of medical residency. Preference for online social media communication was correlated with lower general health concurrently and retrospectively as well as with low self-esteem concurrently. They posit two explanations for the observed association, one problematic usage of the internet having an impact on mental health of medical residents and the other considering online usage as a coping strategy against depression and low self-esteem. In a similar vein, another original cross-sectional investigation looked that the burn out effects that social media overuse can have on vocational settings: Han et al. found a significant positive correlation between social media addiction and job burnout that is either moderated or mediated by social comparison depending on whether the milieu is low or high on social comparison, respectively. While social comparison alone did not correlate with job burnout, social media users who often compare themselves to peers who are worse off in life and feel positive emotions were found to be more prone to job burnout. A study by Yu and Luo found that social media addiction in university students was associated with longer sleeping latency, more sleep disturbance, poorer academic performance, lower levels of life satisfaction, and higher levels of depression with parental reactive restriction and limiting online behaviors showing little effect.

Finally, a systematic review and meta-analysis by Dahl and Bergmark highlighted the deficits in the assessment and natural history of online addiction and internet gaming disorder; while 1-year persistence was demonstrated in about half of the samples of quantitative synthesis, the field was shown generally lacking of adequate data to robustly characterize longitudinal outcomes.

In conclusion, the studies included in this Research Topic drew the wider context of online addiction; while the main focus of research inevitably are the psychological correlates that could culminate up to a psychiatric condition, it is important to keep in mind that this entity is all-encompassing just as the use of digital technologies has an impact on all of our daytime activities.

## Author Contributions

All authors listed have made a substantial, direct and intellectual contribution to the work, and approved it for publication.

## Conflict of Interest

The authors declare that the research was conducted in the absence of any commercial or financial relationships that could be construed as a potential conflict of interest.
